# Biological sex does not predict glymphatic influx in healthy young, middle aged or old mice

**DOI:** 10.1038/s41598-020-72621-3

**Published:** 2020-09-30

**Authors:** Michael Giannetto, Maosheng Xia, Frederik Filip Stæger, Tanner Metcalfe, Hanna S. Vinitsky, Juliana A. M. L. Dang, Anna L. R. Xavier, Benjamin T. Kress, Maiken Nedergaard, Lauren M. Hablitz

**Affiliations:** 1 Center for Translational Neuromedicine, University of Rochester Medical Center, Rochester, NY 14642 USA; 2 Center for Basic and Translational Neuroscience, Faculty of Health and Medical Sciences, University of Copenhagen, 2200 Copenhagen, Denmark; 3 Laboratory of Brain Metabolic Diseases, Institute of Metabolic Disease Research and Drug Development, China Medical University, No. 77, Puhe Street, Shenbei District, Shenyang, 110177 People’s Republic of China; 4 Department of Orthopaedics, The First Hospital of China Medical University, Shenyang, People’s Republic of China

**Keywords:** Circadian rhythms and sleep, Cognitive ageing, Diseases of the nervous system, Glial biology, Neuroimmunology, Sexual dimorphism

## Abstract

Sexual dimorphism is evident in brain structure, size, and function throughout multiple species. Here, we tested whether cerebrospinal fluid entry into the glymphatic system, a network of perivascular fluid transport that clears metabolic waste from the brain, was altered between male and female mice. We analyze glymphatic influx in 244 young reproductive age (2–4 months) C57BL/6 mice. We found no male/female differences in total influx under anesthesia, or across the anterior/posterior axis of the brain. Circadian-dependent changes in glymphatic influx under ketamine/xylazine anesthesia were not altered by sex. This was not true for diurnal rhythms under pentobarbital and avertin, but both still showed daily oscillations independent of biological sex. Finally, although glymphatic influx decreases with age there was no sex difference in total influx or subregion-dependent tracer distribution in 17 middle aged (9–10 months) and 36 old (22–24 months) mice. Overall, in healthy adult C57BL/6 mice we could not detect male/female differences in glymphatic influx. This finding contrasts the gender differences in common neurodegenerative diseases. We propose that additional sex-dependent co-morbidities, such as chronic stress, protein misfolding, traumatic brain injury or other pathological mechanisms may explain the increased risk for developing proteinopathies rather than pre-existing suppression of glymphatic influx.

## Introduction

The brain parenchyma has no traditional lymphatic system. Instead, it has a glial-lymphatic system where astrocytes line perivascular spaces to enable cerebral spinal fluid (CSF) into and interstitial fluid out of the brain, clearing waste^1, 2^. The glymphatic system is highly dependent on cardiovascular function, specifically arterial pulsation^3–5^ and heart rate^6^. Influx of CSF to the brain is increased during sleep^7, 8^, during the day in nocturnal animals^9^, and under certain anesthetic conditions^6^. Although the understanding of the glymphatic system is rapidly expanding, it has garnered controversy due to lack of fundamental knowledge of how basic physiology influences brain fluid transport.

In humans, cardiovascular disease is the number one cause of death for women^10^. Prevalence of Alzheimer’s disease is estimated to be twice as high in females, with a faster rate of cognitive decline after diagnosis of mild cognitive impairment compared to men, and higher rates of brain atrophy^11^, persisting in some transgenic mouse models^12^. These differences may be caused by underlying differences in genetic susceptibility, and lifestyle resilience between men and women^13^. Additionally, there is some evidence in humans^14^ and from rodent models that male and female animals recover differently after traumatic brain injury, with female mice having reduced neuroinflammation^15, 16^ and male mice having increased angiogenesis a week after injury^17^. How sex may affect the glymphatic system, which is impaired in aging^18^, neurodegenerative disease models^19–22^ and traumatic brain injury^21, 23^, remains unknown.

Here, we tested the hypothesis that glymphatic influx is regulated by biological sex by reanalyzing glymphatic influx under six different anesthetics^6^ and six time points across a 24 h day^9^, together with an entirely new dataset collected under the same conditions in middle (9–10 months) and advanced aged (22–24 months) mice. Out of a total of 291 mice, 133 females, we can detect no difference in CSF tracer distribution between male and female mice, independent of anesthetic, time-of-day, anterior to posterior brain slice, brain sub-region, or age.

## Results

### Sex does not predict CSF tracer distribution under anesthesia

**Figure 1:**
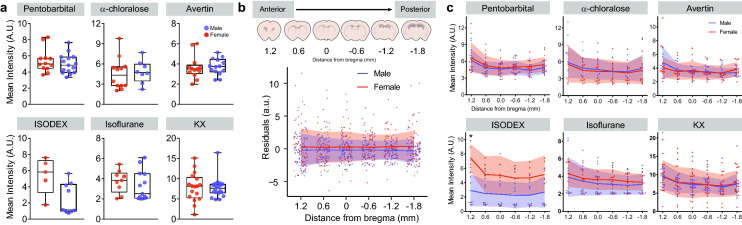
Biological sex has no detectible effect on CSF influx regardless of anesthetic. (**a**) Min/max boxplot of average intensity per animal for male and female mice under six different anesthetics. Individual animals are represented by single, colored dots. Red: female, blue: male. Isoflurane + dexmedetomidine: ISODEX, ketamine/xylazine: KX. (**b**) Anterior to posterior coronal section analysis (top), and residual intensity plots for all mice in every group across six coronal sections (bottom). Residual = (single animal mean fluorescence – mean anesthetic group fluorescence)/total variance in anesthetic group. Single animals are represented by dots. Group mean indicated by solid line. Standard deviation shown by shading. Red: female, blue: male. n = 68 female and 81 male mice (**c**) Mean intensity of fluorescence across 6 coronal sections in male and female mice under six different anesthetics. Mean and standard deviation shown with solid line and shading. Asterisk indicates 2-sided t-test with Bonferroni adjustment: p = 0.015. Individual mice shown by colored dots. Pentobarbital: n = 12 female and 15 male mice; α-chloralose: n = 12 female and 8 male mice; Avertin: n = 13 female and 14 male mice, ISODEX: n = 5 female and 9 male mice; Isoflurane: n = 9 female and 15 male mice, KX: n = 16 female and 20 male mice. Slice cartoons in (**b**) were created by Dan Xue within the Nedergaard lab.

First, we asked whether anesthesia may differentially alter anterior/posterior distribution of CSF tracer in the brain in male and female C57BL6 mice. Fluorescent-tagged bovine serum albumin was injected into the CSF pool in the cisterna magna (CM) and allowed to circulate for 30 min before brain collection and tracer distribution analysis. Brains were sliced into 100 µm coronal sections from 1.2 to − 1.8 mm from bregma, with one slice taken every 600 µm. As expected, anesthesia impacted influx (2-way ANOVA, main effect of anesthesia: F(5,134) = 29.474, p < 0.001; Fig. 1a). We found no male/female difference in tracer distribution (2-way ANOVA, main effect of sex: F(1,134) = 1.883, p = 0.172; sex-by-anesthesia interaction: F(5,134) = 0.383, p = 0.860). When organized by specific anesthetic, ketamine/xylazine (KX), α-chloralose, avertin, isoflurane, and pentobarbital (pento) showed no male/female difference across the anterior/posterior axis (Fig. 1b,c t-test with Bonferroni adjustment: p > 0.24 for all male/female comparisons between slices). For animals anesthetized with isoflurane and dexmedetomidine (ISODEX), females had a trend towards higher influx, though this was non-significant (ISODEX male/female difference: p = 0.157), and had significantly increased tracer in the most anterior slice (t-test with Bonferroni adjustment: p = 0.015). These results could be due to a lower sample size of females compared to males and other anesthetic comparisons (n = 5 female and 9 male mice compared to an average n = 15 mice per group). The conclusion that no difference between CSF tracer influxes between sexes exists was supported by plotting the residuals, where there is clearly no change in influx across slices (Fig. 1b). Biological sex did not improve a linear model (F (6,139) = 25.44, p < 0.001, R^2^ = 0.503, sex coefficient: p = 0.1674). We used forward and backwards stepwise model selections with Akaike's information criterion (AIC) and Bayesian information criterion (BIC) (see “Methods”and^24^). We observed no improvement in BIC when including biological sex, and only a slightly better (from 173.56 to 173.55) score with AIC. This is in contrast to including anesthesia which changes AIC from 269 to 173.56. We conclude that glymphatic influx in anesthetized, 2–4 month-old mice has no detectible dependence on biological sex.

### Anesthesia differentially affects male and female influx rhythms

In humans, females tend to have shorter circadian free-running periods than males by approximately 6 min/day^25^. Additionally, rodent circadian wheel-running behavior changes over the estrous cycle^26–29^. Previous work has shown glymphatic influx has an endogenous, circadian rhythm that peaks during the rest phase of mice^9^. Using data where rhythms in influx were defined via time courses taken every 4 h across the day under three different anesthetic paradigms (KX, pento, and avertin)^9^, we next tested whether there was a sex difference in glymphatic influx across the day.

**Figure 2:**
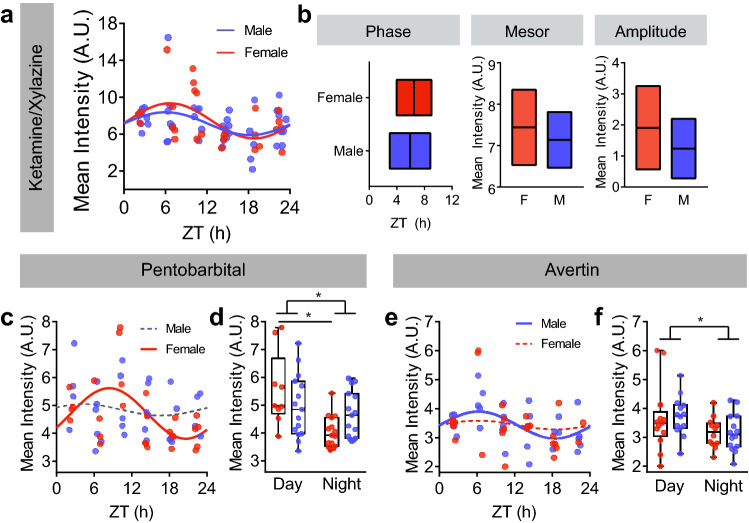
Male/female differences in influx rhythms. (**a**) Mean average intensity per animal over time under ketamine/xylazine anesthesia. Individual animals are colored dots, solid lines indicate significant cosinor fit. Red: female, blue: male. ZT: zeitgeber time, where ZT0 is lights on, ZT12 lights off. n = 29 female and 42 male mice, 4–9 mice per time point. (**b**) Comparison of 95% confidence intervals for phase, mesor, and amplitude as predicted by cosinor analysis under ketamine/xylazine anesthesia for male (blue) and female (red) animals. Midline: cosinor estimate, boxes: upper and lower 95% confidence interval boundaries. (**c**) Mean average intensity per animal over time under pentobarbital anesthesia. Individual animals are colored dots, solid lines indicate significant cosinor fit, dotted lines indicate insignificant fit. For cd: n = 27 female (12 day, 15 night) and 29 male (14 day, 15 night) mice; 4–6 animals per time point; (**d**) Min/max boxplot of average intensity during the day and night between male and female animals under pentobarbital anesthesia. Minima: minimum value, maxima: maximum value, center: median, quartiles: box and whisters. *p < 0.05. Individual mice represented by colored dots. (**e**) Mean average intensity per animal over time under avertin anesthesia. Individual animals are colored dots, solid lines indicate significant cosinor fit, dotted lines indicate insignificant fit. n = 25 female (13 day, 12 night) and 28 male (14 day, 14 night) mice, 2–6 per time point. (**f**) Min/max boxplot of average intensity during the day and night between male and female animals under avertin anesthesia. Minima: minimum value, maxima: maximum value, center: median, quartiles: box and whisters. *p < 0.05. Individual mice represented by colored dots.

Under KX, cosinor analysis for male (n = 42) and female (n = 29) mice revealed significant rhythms in both groups (Male: F(3,39) = 3.427, p = 0.043, R^2^ = 0.149; Female: F(3,26) = 4.376, p = 0.023, R^2^ = 0.252; Fig. 2a). We found no significant difference in mesor, amplitude, or phase of glymphatic influx rhythms when comparing 95% confidence intervals (Fig. 2b). For both pento (males: n = 29 mice, females: n = 27 mice) and avertin (males: n = 28 mice, females: n = 25 mice), cosinor analysis resulted in differential effects based on sex (Fig. 2c,e). Males anesthetized with pento had no detectible rhythm in CSF influx (F(2,27) = 0.317, p = 0.731), whereas females had a rhythm (F(2,24) = 4.683, p = 0.019, R^2^ = 0.281) peaking at Zeitgeber Time (ZT; ZT 0 lights on, ZT 12 lights off) 8.25. Under avertin, females had no significant rhythm (F(3,22) = 2.139, p = 0.142), and males had a significant rhythm (F(2,25) = 3.464, p = 0.047, R2 = 0.217) peaking at ZT 6.13. Confidence interval estimates for peak timing overlapped in all rhythmic groups.

Because the cosinor analysis fits the data to a linearized sine function, it is a parametric test that assumes equal variance and similar sampling across the day. The male/female difference in cosinor analyses could be due to insufficient sample sizes across time under pentobarbital and avertin, which have smaller sample sizes overall. To address this concern, we pooled data points between the day (ZT 2–11) and night (ZT 12–22) and compared between male and female animals. For both pentobarbital and avertin, there was a main effect of time-of-day (pentobarbital: 2-way ANOVA, F(1,50) = 11.49, p = 0.0014; avertin: F(1,49) = 5.767, p = 0.0202; Fig. 2d). There was a significant sex-by-time interaction in the pentobarbital group (2-way ANOVA, F(1,50) = 4.995, p = 0.0299; Fig. 2f), though it was driven by day/night differences in the females (Tukey’s posthoc, p = 0.0028, all other comparisons: p > 0.05). Overall, there was no significant main effect of sex on day/night differences in glymphatic influx.

### Age does not reveal a male/female difference in CSF tracer distribution

**Figure 3:**
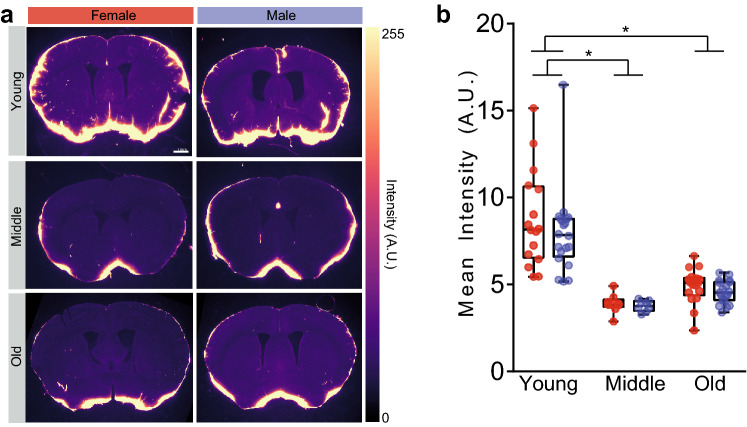
Glymphatic influx decreases with age independent of sex. (**a**) Representative coronal sections at 0.6 mm anterior to bregma for young (2–4 months), middle (9–10 months), and old (22–24 months) male and female mice under KX anesthesia. White scale bar is 1 mm. Color intensity scale on the right in arbitrary units (A.U.). (**b**) Min/max boxplots of mean average fluorescent intensity. Individual animals represented by colored dots. Red: female, blue: male. Asterisk indicates p < 0.005. Young: n = 16 female and 20 male mice. Middle: n = 9 female and 8 male mice. Old: n = 17 female and 19 male mice.

**Figure 4:**
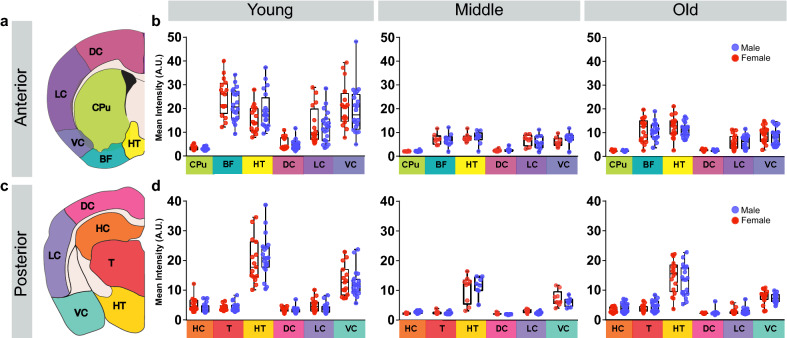
Aged animals have no detectible sex differences in tracer subregion distribution. (**a**) Schematic of subregions where fluorescence was calculated in anterior (0.6 mm from bregma) coronal sections. *DC* Dorsal cortex, *LC* lateral cortex, *VC* ventral cortex, *CPu* caudate putamen, *BF* basal forebrain, *HT* hypothalamus. (**b**) Min/max boxplots of mean subregion intensity in young, middle and old animals. Subregions also color coded to (**a**). Individual animals represented by colored dots. Red: female, blue: male. (**c**) Schematic of subregions where fluorescence was calculated in posterior (− 1.8 mm from bregma) coronal sections. *DC* Dorsal cortex, *LC* lateral cortex, *VC* ventral cortex, *HC* hippocampus, *T* thalamus, *HT* hypothalamus. (**d**) Min/max boxplots of mean subregion intensity in young, middle and old animals. Subregions color coded to (**d**). Individual animals represented by colored dots. Red: female, blue: male. Young: n = 16 female and 20 male mice. Middle: n = 9 female and 8 male mice. Old: n = 17 female and 19 male mice. Slice schematics in (**a**) and (**c**) were created by Dan Xue within the Nedergaard lab.

We were unable to detect significant effects of sex on glymphatic influx on 2–4 month old animals. Next, we sought to test the hypothesis that as the glymphatic system is challenged, sex differences may be exacerbated. Glymphatic influx declines with age^18^ when cardiovascular and neurodegenerative disease risk increases. Females are at greater risk for developing both cardiovascular disease and Alzheimer’s disease^10, 11, 13^. We next asked whether changes in glymphatic influx in middle aged (9–10 months) and old (22–24 months) animals were dependent on biological sex. Then, we compared CSF tracer distribution in different sub-regions in anterior and posterior coronal sections. Old (n = 26 total mice) and middle-aged (n = 17 total mice) mice were anesthetized with KX and were only compared to the young mice anesthetized with KX (n = 36 total mice). Similar to previous findings, average CSF tracer influx was lower in older animals than younger animals (Kruskal–Wallis test, H(6) = 65.26, p < 0.0001; all comparisons of young to middle or old: Dunn’s multiple comparisons test, p < 0.005; all comparisons of middle to old: Dunn’s multiple comparisons test, p > 0.35; Fig. 3) with no effect of sex (female/male young, female/male middle, and female/male old: Dunn’s multiple comparisons test, p > 0.9999). Consistent with this finding younger animals had increased influx compared to middle aged and old animals in all measured sub-regions (Fig. 4). There were no detectable male/female differences in tracer distribution among any sub regions at any age (t-test with Bonferroni adjustment: p > 0.43 for all comparisons). There is a visual, though not statistically significant, trend for middle aged animals to have lower influx than the old cohort. We speculate this may be due to remodeling of perivascular spaces with age. Because these animals were rapidly decapitated, brains harvested within 2 min and the brains drop-fixed instead of perfusion-fixed, we are confident the decreased glymphatic influx observed with aging is independent of any perfusion artifacts^3^ or effects of cardiac arrest^30^ that may have skewed previous results^18^, where this trend was not detected.

## Discussion

We used a dataset of over 230 C57BL/6 animals to determine if biological sex was a predictive component of CSF influx into the murine brain. We found no significant male/female difference in anterior to posterior CSF tracer distribution under six different anesthesia, though ISODEX co-treatment had a trend toward increased influx in female mice. Under KX anesthesia there is no difference in daily circadian rhythmicity of CSF influx between females and male, though pentobarbital and avertin may have sex-dependent influences on rhythmicity of influx. Finally, we show there is no male/female difference in total influx, or region specific influx in cohorts of aged animals. These results support the conclusion that in healthy animals, biological sex does not alter basal CSF influx into the brain. This observation is surprising taking the marked sex differences in age-dependent loss of cognitive function, proteinopathies, cardiovascular diseases, inflammation and more into consideration^10–13, 16, 31–33^.

Sexual dimorphism in brain size could partially explain the lack of significant differences in CSF tracer influx. In mice, males tend to have increased body weight compared to females. Yet, data from Jackson Labs on C57BL6 mice shows less than 1% difference in total brain weight from over 100 animals (phenome.jax.org). Using magnetic resonance imaging, a separate report showed brain volume changes of less than 2.5% difference between male and female mice^34^. Another study used postmortem 3D reconstruction, and estimated no difference in brain volume or surface area between male and female mice^35^. Finally, if there were subtle differences in vascular and perivascular structure they should be emphasized with aging when these systems have been taxed throughout the life of the animal and therefore begin breaking down in different ways. Even with aging, we found no differences in glymphatic influx between middle aged (9–10 months) or extremely old (22–24 month) male and female mice. Taken together, we conclude that overall changes in brain size or vascular structure did not skew our results.

Female estrous can influence neuronal function^33^ and circadian behavior^25–29^, indicating it could contribute to variability in CSF influx along perivascular spaces into the brain. The experiments used for these analyses split data collection over many days to randomize estrous states. Although we found no increased variability of female data, which suggests that estrous does not significantly influence glymphatic influx in normal, adult females, more work must be done to explicitly control for estrous state. Future studies may also investigate how large-scale hormonal changes in female animals, such as during adolescence, pregnancy, post-pregnancy, or post sexual senescence influence the glymphatic system.

In healthy animals we find no effects of biological sex on glymphatic influx across anesthesia, the day, and in aging. This was unexpected because we know sex affects common disorders, such as cardiovascular disease prevalence^10^, Alzheimer’s disease^11, 13, 36^, and recovery from traumatic brain injury^15–17^, which generally correlate to glymphatic dysfunction. The observations reported here suggest it is not underlying male/female differences in glymphatic function that drive disease, but rather differences in homeostatic responses to disease that lead to male/female differences in pathology, prevalence, and long-term recovery. If glymphatic function had intrinsic male/female differences, we expect sex ratios of diseases where glymphatic clearance has been implicated in the pathology, such as AD and Parkinson’s disease^19–22^, to be the same. However this is not the case; females are at higher risk for Alzheimer’s disease^11, 12^ and males are at higher risk for Parkinson’s disease^36^. One intriguing possibility is that chronic stress, which in rodent models can alter neuronal morphology and cognition differentially between sexes^31, 33, 37, 38^, accumulates over time and these life-time stressors may alter glymphatic flow in a sex-dependent manner.

## Methods

### Animals

All experiments were approved and in accordance with relevant guidelines and regulations by the University of Rochester Medical Center Committee on Animal Resources, certified by Association for Assessment and Accreditation of Laboratory Animal Care. C57BL/6 mice ages 2–4 months (25–30 g) were purchased from Charles River Laboratories (Wilmington, MA). Mice were group-housed in a 12:12 h light/dark cycle with ad libitum access to food and water. Experiments were done over several consecutive days, to randomize phase of the female estrous cycle. For anesthesia and aging experiments, all data was collected during the light phase. All efforts were made to keep animal usage to a minimum. Two cohorts of animals were taken from^6^ and^9^ to analyze the effects of anesthesia and circadian-timing on glymphatic influx, respectively. Aged C57BL/6 mice were procured from the U.S. National Institute on Aging, and were 22–24 months old.

### Anesthesia

All anesthesia was administered as in Hablitz et al.^6^ Anesthesia was administered as follows: a mixture of racemic ketamine (100 mg/kg) and xylazine (20 mg/kg) intraperitoneally (ip), pentobarbital (60 mg/kg ip), 2,2,2-tribromoethanol (also known as Avertin; 120 mg/kg ip), α-chloralose (80 mg/kg ip), and ISO (initial induction at 4%, maintained at 1 to 2% for the duration of the experiment). For the α-chloralose regimen, surgery was initially done under 1 to 2% ISO and maintained at 0.5% ISO following CM injection because the drug is technically a hypnotic rather than a general anesthetic. In a separate cohort of mice, dexmedetomidine was given as a supplement to ISO induction at 0.015 mg/kg ip, with a second equal-sized bolus administered just before the 30-min tracer circulation period. ISO was always delivered with 100% oxygen. Directly before CM infusion, the animal received an additional one-tenth of the initial dosage, and the pedal reflex was tested every 5 to 10 min during the tracer circulation time to ensure adequate anesthetic depth throughout the study.

### Intracisternal CSF tracer infusion

Cisterna magna (CM) injections were performed as described previously^3, 6, 9, 39^. In brief: fluorescent CSF tracer (bovine serum albumin, Alexa Fluor 647 conjugate; 66 kDa; Invitrogen, Eugene, OR) was formulated in artificial CSF at a concentration of 0.5% w/v. Anesthetized mice were fixed in a stereotaxic frame, the CM surgically exposed, and a 30 gauge needle connected to PE10 tubing filled with the tracer was inserted into the CM. Ten microliters of CSF tracer was infused at a rate of 2 μl/min for 5 min with a syringe pump (Harvard Apparatus). To visualize tracer movement from the cisternal compartments into the brain parenchyma, the animals were killed by decapitation and the brain removed 30 min after the start of intracisternal infusion (note that the needle was left in place after the infusion to prevent backflow of CSF). The brain was fixed overnight by immersion in 4% paraformaldehyde in PBS. Coronal vibratome slices (100 μm) were cut and mounted. Tracer influx into the brain was imaged ex vivo by macroscopic whole-brain and whole-slice conventional fluorescence microscopy with identical acquisition parameters across groups (Olympus; Stereo Investigator Software).

Tracer influx was quantified by a blinded investigator using ImageJ 1.52i as described previously^6^. The cerebral cortex in each slice was manually outlined, and the mean fluorescence intensity within the cortical ROIs was measured. An average of fluorescence intensity was calculated between six slices for a single animal, resulting in a single biological replicate. Equivalent slices were used for all biological replicates.

### Statistical analysis

For testing effects of anesthesia and sex on glymphatic influx in both average slice-fluorescence and across 6 anterior/posterior slices, we used a 2-way ANOVA, followed by Bonferroni P value correction for multiple comparisons. Next, we fit the data to a general linear model, and tested the fit of the model using Akaike's information criterion (AIC) and Bayesian information criterion (BIC) with and without the inclusion of sex as a covariate. For a discussion of residual comparison, compared to AIC, compared to BIC please see: Vrieze et al.^24^.These statistical analyses were performed in R version 3.5.2 (2018–12–20). When testing for sex differences in rhythmicity of influx across three anesthetics, Cosinor analyses^40, 41^ were performed using PASW Statistics 18. For comparisons of means in samples with normal distributions and homogeneous variances (as indicated by a Levene’s test), ANOVA was used for comparisons between two or more means, followed by a Tukey multiple comparisons test. Age by sex interactions were tested using a Kruskal–Wallis test followed by Dunn’s multiple comparisons tests due to unequal sample size in the middle-aged group compared to young and old. These tests were performed in GraphPad Prism 7.0d. For comparisons of means in samples with normal distributions and homogeneous variances (as indicated by a Levene’s test), an ANOVA was used for comparisons between subregions, followed by Bonferroni p value correction for multiple comparisons in R version 3.5.2 (2018–12–20). For all experiments, significance was ascribed at p < 0.05.

## Data Availability

All data needed to evaluate the conclusions in the paper are present in the paper. Additional data available from authors upon request.
